# LINC00261 elevation inhibits angiogenesis and cell cycle progression of pancreatic cancer cells by upregulating SCP2 via targeting FOXP3

**DOI:** 10.1111/jcmm.16930

**Published:** 2021-09-19

**Authors:** Jun Zou, Xuanzeng Pei, Dan Xing, Xiaojun Wu, Shuai Chen

**Affiliations:** ^1^ Department of abdominal surgery Jiangxi Cancer Hospital Nanchang China; ^2^ Department of General Surgery Affiliated Hospital of Jiaxing University Jiaxing China

**Keywords:** angiogenesis, cell cycle progression, forkhead box P3, long non‐coding RNA LINC00261, pancreatic cancer, sterol carrier protein‐2

## Abstract

Long non‐coding RNAs (lncRNAs) biological functions and molecular mechanisms associated with pancreatic cancer (PC) remain to be poorly elucidated. We aimed to clarify the role of lncRNA LINC00261 (LINC00261) in PC and confirm its regulatory mechanisms. Bioinformatics analysis, RNA pull‐down and RIP assays were performed to investigate relationship between LINC00261 and forkhead box P3 (FOXP3). Further, dual‐luciferase reporter gene and ChIP assays were employed to confirm the relationship among LINC00261, FOXP3 and sterol carrier protein‐2 (SCP2). PC cells were introduced with a series of vectors to verify the effects of LINC00261 and SCP2 on the viability, cell cycle progression, migration and angiogenesis of PC cells. Nude mice with the xenograft tumour were used to evaluate the effects LINC00261 on the tumourigenicity. LINC00261 was lowly expressed in PC tissues and cells. SCP2 was inhibited by LINC00261 through FOXP3. Functionally, upregulated LINC00261 or downregulated SCP2 led to reduced cell viability, migration, angiogenesis and tumourigenicity potentials. This study demonstrated the inhibitory role of LINC00261 in the angiogenesis and cell cycle progression of PC cells. It acts through the negative regulation of SCP2 via targeting FOXP3. Findings in this study highlight a potentially biomarker for PC treatment.

## 1 INTRODUCTION

Pancreatic cancer (PC) is one of the most aggressive and malignant tumours with a dismal five‐year survival rate.^1^, ^2^, ^3^ Indeed, over 80% of PC patients already have metastasis or locally advanced tumours at time of diagnosis, and less than 15%–20% of patients are suitable for surgical resection^4^, ^5^. The poor prognosis of PC is largely attributable to its late diagnosis,^6^, ^7^, ^8^ which implies that identification of specific diagnostic markers might improve the outcome.

Long non‐coding RNAs (lncRNAs) serve as key mediators in many cellular processes.^9^ For example, decreased LINC00261 expression occurs in gastric cancer, whereas its over‐expression could inhibit cell growth dynamics.^10^, ^11^ Moreover, Chen et al.^12^ found that LINC00261 was also associated with PC cell migration. Through a search of the MEM database, we observed that sterol carrier protein‐2 (SCP2) expression is likely to be regulated by LINC00261. SCP2, a nonspecific lipid‐transfer protein, exhibits potential functions in intracellular lipid transport and metabolism and is furthermore associated with diseases related to lipid trafficking abnormalities.^13^ Notably, SCP2 is capable of regulating angiogenesis and tumour migration.^14^ Forkheadbox protein 3 (FOXP3), a pivotal transcription factor for regulation of T cells, has been identified as a key regulator in pancreatic ductal adenocarcinoma.^15^ Furthermore, FXOP3 has a regulatory role in the migration of breast cancer cells,^16^ suggesting a general involvement in neoplastic progression. Meanwhile, FOXP3 is reportedly under the fine expressional control from lncRNA EGFR‐AS1 in the context of non‐small cell lung cancer (NSCLC) progression.^17^ In the present study, we used prediction and enrichment analysis to determine the target relationships among LINC00261, FOXP3 and SCP2 and uncover their function in PC using in vitro and in vivo experiments.

## 2 MATERIALS AND METHODS

### 2.1 Ethics statement

This study was granted by the Ethics Committee of The Affiliated Hospital of Jiaxing University. Informed consent was obtained from all patients. All animal experiments were approved by the Animal Ethics Committee of The Affiliated Hospital of Jiaxing University. Great efforts were made to minimize the number of animals used in the experiments and their discomfort.

### 2.2 Bioinformatic prediction

The PC‐related gene expression profiles (GSE16515 and GSE27890) and annotated probe files were downloaded from the Gene Expression Omnibus (GEO) database (http://www.ncbi.nlm.nih.gov/geo) followed by analysis through the Affymetrix Human Genome U133 Plus 2.0 Array. GSE16515 includes 16 normal samples and 36 tumour samples, and GSE27890 includes 4 normal samples and 4 tumour samples. With the normal samples as controls, we used the Affy installation package of the R software for background correction and normalization of the microarray data.^18^ Then, we applied the linear model, empirical Bayesian statistics method of the Limma installation package combined with a traditional *t*‐test for non‐specific screening of data to identify differentially expressed lncRNAs^19^ via the Multi Experiment Matrix website (MEM, http://biit.cs.ut.ee/mem/).^20^ Finally, the Web‐based Gene Set Enrichment Analysis Toolkit (WebGestalt; http://www.webgestalt.org) was adopted to perform Kyoto Encyclopedia of Genes and Genomes (KEGG) pathway enrichment analysis.^21^

### 2.3 Sample collection

Pancreatic cancer tissues were collected from 57 PC patients (34 males and 23 females, average age: 58 years) who underwent pancreatectomy in the Affiliated Hospital of Jiaxing University from January 2014 to December 2017. Adjacent normal tissues were collected as the controls. Among all patients, 9 cases had tumour size ≤2 cm, 21 had tumour size 2–4 cm, and 27 cases had tumour size >4 cm. According to the tumour node metastasis (TNM) stages,^22^ 18 cases were classified to stage I, 27 cases were stage II, 12 cases were stage III, and 30 cases were diagnosed with lymph node metastasis (LNM). A serial section from each specimen was stained with haematoxylin & eosin staining for histological evaluation.^23^ Histological examination showed that 24 cases were highly differentiated, 20 cases were moderately differentiated, and 13 cases were poorly differentiated. Besides, 24 of the 57 cases were diagnosed with venous invasion and 33 cases without venous invasion. The patients meeting following criteria were included: (1) patients with PC were confirmed both by tumour pathology and genetics; (2) tumours were clinically evaluated to be amenable to surgical resection; (3) patients were fit to tolerate abdominal surgery; (4) patients didn't present with complications of other gastrointestinal tumours or history of other tumours; (5) chemotherapy or radiotherapy had not been performed before surgery. Patients with severe impairment in heart, liver, kidney and other organs, a history of autoimmune disease, in the active stage of a chronic infectious disease or diagnosis of an acute infectious disease were excluded. The serum of PC patients and 57 healthy people was collected, where the expression of LINC00261 in the serum was detected by enzyme‐linked immune‐sorbent assay (ELISA).

### 2.4 ELISA

According to instructions, Quantikine ELISA Human Galectin‐9 immunoassay kit (DGAL90, R&D Systems) was used to determine LINC00261 expression in serum of PC patients and healthy donors.

### 2.5 Reverse transcription quantitative polymerase chain reaction (RT‐qPCR)

Pancreatic cancer tissues and adjacent normal tissues (30 mg each) were isolated for total RNA determination using the Trizol kit (Invitrogen). PrimeScript^™^ RT reagent Kit (RR047A, Beijing Think‐Far) was used to reversely transcribe RNA into cDNA (50 ng/μl). Primers were designed by the Premier 5.0 software and synthesized by TsingKe (Table S1). ABI 7900HT real‐time quantitative PCR instrument (ABI 7900, PuDi) was applied for analysis, with glyceraldehyde‐3‐phosphate dehydrogenase (GAPDH) and U6 as internal controls.

### 2.6 Western blot analysis

Western blot analysis was performed with following antibodies (all from Abcam)^24^: anti‐rabbit antibodies to FOXP3 (ab215206), SCP2 (ab140126), vascular endothelial growth factor (VEGF; ab10766), Ki67 (ab92742), CyclinD1 (ab16663) and Brachyury (ab209665) as well as horseradish peroxidase (HRP)‐labelled goat anti‐rabbit antibody (1: 5000). After washing, enhanced chemiluminescence was used to develop images. The optical density (OD) value of the protein bands was measured by a gel imaging analysis system with relative protein content as the ratio of average OD value of the target to that of the internal control.

### 2.7 Cell culture and transfection

Human pancreatic ductal epithelial cell line H6c7 and human PC cell lines SW1990 and PANC‐1 (American Type Culture Collection) were cultured in RPMI 1640 culture medium (22400089, Gibco BRL.) containing 15% foetal bovine serum (FBS) and seeded into 6‐well plates (2.5 × 10^5^/well) for incubation. The culture medium was renewed every 2–3 days based on the growing states of cells. Cells were sub‐cultured upon 80%–90% cell confluence.

Cells (logarithmic growth phase) were seeded into a 6‐well cell plate (2 × 10^5^ cells/well) for further culture and transfected using a Lipofectamine 2000 kit (Invitrogen) upon 90% cell confluence. Cells were transfected with Vector, overexpressed (oe)‐LINC00261, shRNA (sh)‐control, sh‐LINC00261, oe‐LINC00261 + Vector, LINC00261 + sh‐negative control (NC), LINC00261 + FOXP3, LINC00261 + sh‐SCP2, respectively. Briefly, 5 μl lipofectamine 2000 was mixed with 250 μl serum‐free medium Opti‐MEM and incubated at ambient temperature (5 min). The mixture was incubated at ambient temperature for 20 min and added to the cell culture well (37℃). After culture for 6–8 h under 5% CO_2_ conditions, the complete medium was renewed, and cells were cultured for 24–48 h for subsequent experiments. The primer sequence for sh‐SCP2: forward: AGAGAACTTTCTTCATGCTATTCGGGGTGTTTCGTCCTTTCCA; reverse: CTAAAAACCGAATAGCATGAGATAGAAGAGAACTT; sh‐RNA: forward: AGAGAACTTACTATTCACCAACCTCCTCGGTGTTTCGTCCTTTCCA; reverse: GGAATTCAAAAAGAGGAGCTTGGTGAATAGTAGAGAACTT.

### 2.8 Dual‐luciferase reporter gene assay

Binding site of FOXP3 in promoter region of SCP2 was investigated by Website analysis prediction. The SCP2 promoter region was constructed into the pGL3‐Basic vector (Promega) as a recombinant vector SCP2‐wild type (WT), and the FOXP3 binding site of SCP2 was mutated into the pGL3‐Basic vector (Promega) as a recombinant vector SCP2‐mutant (MUT). The sequenced luciferase reporter plasmids SCP2‐WT and SCP2‐MUT were co‐transfected into HEK‐293T cells with sh‐NC, sh‐FOXP3, oe‐NC and oe‐FOXP3, respectively. Following 48‐h transfection, luciferase activity was measured using a luciferase detection kit (K801‐200, Biovision) and Glomax20/20 luminometer fluorescence detector (Promega).

### 2.9 Fluorescence in situ hybridization (FISH)

Fluorescence in situ hybridization was performed as per the instructions of Ribo™ lncRNA FISH Probe Mix (Red) (RiboBio). Five randomly selected fields of view were photographed under a fluorescence microscope (Olympus).

### 2.10 RNA binding protein immunoprecipitation (RIP) assay

The binding of LINC00261 to the transcription factor FOXP3 was detected using a RIP kit (Millipore)^25^ with the following antibodies: anti‐FOXP3 (sc‐53876, 2 µg per 1 ml of cell lysate, Santa Cruz Biotechnology) and Immunoglobulin G (IgG; ab6785, Abcam, used as NC).

### 2.11 Chromatin Immunoprecipitation (ChIP) assay

SW1990 cells were fixed with formaldehyde (10 min) to generate DNA‐protein cross‐linking, followed by ultrasonic disruption. Cells were centrifuged (4℃; 10 min) under 12,000 *g*, and the collected supernatant was divided into two tubes and incubated with IgG and anti‐FOXP3 (sc‐53876, 2 µg per 1 ml of cell lysate, Santa Cruz) overnight at 4℃, respectively. The protein–protein complexes were precipitated with Protein Agarose/Sepharose. After centrifugation for 5 min, the supernatant was discarded and the non‐specific complexes were washed. De‐crosslinking was then performed at 65℃ overnight. DNA fragment was recovered by extraction and purification with phenol/chloroform. Binding in SCP2 promoters were detected by RT‐qPCR.

### 2.12 Cell counting kit 8 (CCK‐8) assay

Viability of cells was detected by CCK‐8 kit (Beyotime) at 24, 48, 72, and 96 h after seeding. Optical density (OD) value at 450 nm was assessed with a microplate reader.

### 2.13 Flow cytometry

At 48th hour after transfection, cells were trypsinized to adjust the concentration to 1 × 10^6^ cells/ml. Then, 1 ml of cells was centrifuged at 1200 *g* for 10 min. Following supernatant removal, pellets were resuspended in PBS, centrifuged and fixed with pre‐cooled 70% ethanol at 4℃ for overnight incubation. After PBS rinsing, 100 μl of cell suspension was incubated with 50 μg of propidium iodide staining solution (40710ES03, Qcbio) containing RNAase for 30 min in the dark. After the cell suspension was filtered by a 100‐mesh nylon net, the cell cycle was measured by a flow cytometer (Becton, Dickinson and Company) at the excitation wavelength of 488 nm.

### 2.14 Cell colony formation assay

Cells were seeded into a 75‐mm dish (the proportion of single cells in the cell suspension >95%), 800 cells per dish. After 10 day of culture, the cells were rinsed with pH6.8 PBS twice, fixed in methanol for 20 min and coloured with 10% Giemse stain for 20 min. Then, the number of colonies containing over 20 cells per dish was calculated under a dissecting microscope.

### 2.15 Matrigel assay

Human umbilical vein endothelial cells (HUVECs; INS‐1, Shanghai Zishi Biological Technology Co., Ltd.) were cultured with SW1990 cell culture solution in a 24‐well plate (250 μl/well) pre‐coated with pre‐chilled Matrigel. Then, the cells were detached with 1 ml trypsin containing EDTA, and the detachment was stopped by addition of serum‐containing culture solution. After counting, the cells were resuspended and adjusted (1 × 10^5^ cells/ml). After matrigel were solidified, the 24‐well plate was removed, followed by the addition of 500 μl/well of the cell suspension. Afterwards, the plate was incubated for 24 h at 37℃ with 5% CO_2_ and observed under an inverted microscope. Mean number of tubes formed was calculated in three randomly selected fields.

### 2.16 Transwell assay

Migration assay was assessed by 24‐well plate as described previously.^26^ The plates were photographed under an inverted microscope, and the mean number of invaded cells was recorded in 10 randomly selected fields.

### 2.17 Tumour formation in nude mice

A total of 25 nude mice in specific pathogen‐free (SPF) grade were infected with oe‐LINC00261, sh‐LINC00261, oe‐FOXP3, and sh‐SCP2, respectively, by subcutaneous inoculation of stably infected PANC‐1 cells in the right hind leg (1 × 10^6^, 200 μl) (5 mice each). After inoculation, mice were reared under standard conditions, and tumour length and width were measured every 7 days. Size, length (L) and width (W) of subcutaneous transplanted tumour model were measured using Vernier callipers. The volume of tumour was calculated as length × width^2^/2. All mice were euthanized at the 28th day, and the tumour was excised. The excised tumour was then fixed in 10% neutral formalin solution (24 h), dehydrated, embedded and stored for future examination.

### 2.18 Immunohistochemistry (IHC)

Paraffin‐embedded tumour was cut into sections at the thickness of 3 ~ 4 μm. The sections were dewaxed, hydrated, placed in 3% H_2_O_2_ for 10 min and subjected to high‐pressure antigen retrieval for 90 s. Then, the sections were blocked with 5% bovine serum albumin (BSA) for 30 min at 37℃, incubated with 50 μl of rabbit anti‐mouse antibodies to CD31 (1: 100, ab28364, Abcam), VEGF (1: 100, ab2349, Abcam) and Brachyury (1: 100, ab140661, Abcam) overnight at 4℃. PBS was used as the primary antibody in the NC group. The sections were incubated with biotinylated mouse anti‐goat antibody to IgG (50 μl; SF8‐0.3, 1: 100, Solarbio) for 30 min (37℃). Next, the sections were treated with salvianolic acid B and diaminobenzidine solutions for the chromogenic reaction, followed by haematoxylin treatment for 5 min. Later, sections were dehydrated, cleared and mounted. Five randomly selected fields were observed under a light microscope for each section. Positive cells were defined as cells containing ≥25% brownish yellow‐staining located mainly in the cytoplasm, cell membrane or vascular endothelium. The magnitude of microvessel density (MVD) was estimated by the presence of CD31‐positive cells. Endothelial cell clusters or single endothelial cells which shared clear boundaries with the neighbouring tumour cells, microvessel and surrounding connective tissues and presented a brown or brownish yellow colour were deemed to be microvessels. In the condition of disconnected structures, branch structure was counted as one set of microvessels. Five high power visual fields were randomly selected to observe localization of positive expression and quantify positive expression of VEGF and MVD.

### 2.19 Statistical analysis

All data were presented by mean ± standard deviation. SPSS 21.0 software (IBM Corp.) was used for data analysis. Comparison between two groups was analysed by unpaired *t*‐test. Comparisons among multiple groups were analysed using one‐way analysis of variance (ANOVA). *p* < 0.05 was considered statistically significant.

## 3 RESULTS

### 3.1 Downregulation of LINC00261 occurs in PC tissues

**FIGURE 1:**
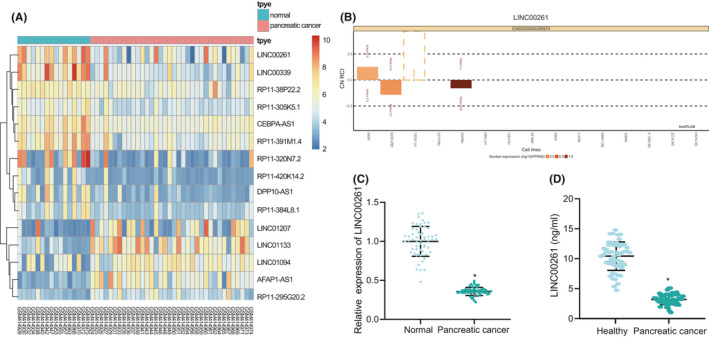
LINC00261 is lowly expressed in PC. (A) LINC00261 expression in the GSE16515 dataset. (B) LINC00261 expression in the GSE27890 dataset. (C) Expression of LINC00261 in PC tissues and adjacent normal tissues determined by RT‐qPCR (*n* = 57). (D) The expression of LINC00261 in the normal pancreatic ductal epithelial cell line H6c7 and two PC cell lines (SW1990 and PANC‐1) measured with RT‐qPCR. *p < 0.05 vs. adjacent normal tissues or normal pancreatic epithelial cells. Comparison between two groups was analysed using the *t*‐test

After screening PC‐related datasets GSE16515 and GSE27890, we found poor expression of LINC00261 (Figure 1A,B). RT‐qPCR showed a notable reduction in the expression of LINC00261 in PC tissues and cells (Figure 1C). ELISA revealed that the expression of LINC00261 was significantly reduced in the serum of PC patients (Figure S1). Next, RT‐qPCR was performed to measure the expression of LINC00261 in the normal pancreatic ductal epithelial cell line H6c7 and two PC cell lines (SW1990 and PANC‐1), which demonstrated that downregulation of LINC00261 also occurred in SW1990 and PANC‐1 cells (Figure 1D).

Moreover, relationships between LINC00261 expression and relevant clinicopathological characteristics of PC patients were explored, revealing that LINC00261 expression had significant correlation with differentiation degree, LNM, venous invasion and TNM stages, whereas no correlation was observed for the gender, age, tumour sites and volume (all *p* > 0.05) (Table S2).

### 3.2 LINC00261 overexpression represses oncogenic phenotype of PC cells

**FIGURE 2:**
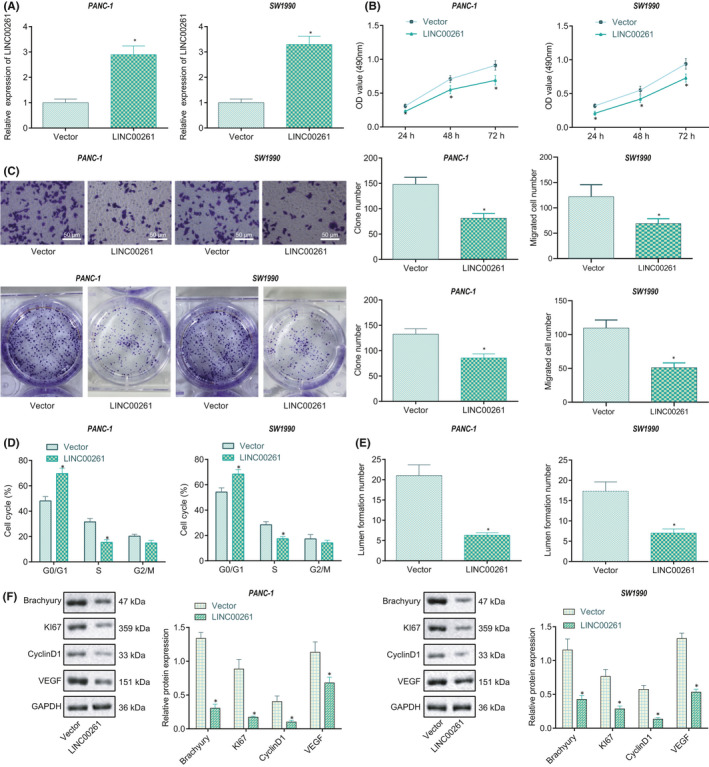
LINC00261 elevation inhibits oncogenic phenotype of PC cells. **(**A) The transfection efficiency of LINC00261 overexpression in SW1990 and PANC‐1 cells after treatment of LINC00261 measured by RT‐qPCR. (B) The effect of LINC00261 overexpression on the cell growth of SW1990 and PANC‐1 cells after treatment with LINC00261 detected by CCK8. (C) Number of clones and cell migration separately assessed by colony formation and Transwell assays, and the corresponding statistics. (D) Cell cycle of SW1990 and PANC‐1 cells after treatment with LINC00261 detected by flow cytometry. (E) Angiogenesis ability of SW1990 and PANC‐1 cells after treatment with LINC00261 detected by Matrigel‐based tube formation assay, and the statistical map. (F) The protein expression of Brachyury, Ki67, Cyclin D1, and VEGF in SW1990 and PANC‐1 cells after treatment with LINC00261 determined by Western blot analysis normalized to GAPDH, and the statistical results. *p < 0.05 compared with cells transfected with Vector. Each experiment was repeated three times

To explore the effect of LINC00261 on the biological features of PC cells, PC cells SW1990 and PANC‐1 were transfected with oe‐LINC00261 or sh‐LINC00261. First, RT‐qPCR confirmed the successful transfection of oe‐LINC00261 and sh‐LINC00261 (Figure 2A; Figure S2A). As indicated by CCK8, Transwell and clone formation assays, over‐expressed LINC00261 inhibited the proliferative, migrative and clone formation capabilities of the cells, while opposite trends were observed in response to sh‐LINC00261 (Figure 2B,C; Figure S2B,C). Besides, flow cytometry results showed that the cells were arrested at the G0/G1 phase, but being reduced in the number at the S phase after overexpression of LINC00261, while opposite trends were observed after knockdown of LINC00261 (Figure 2D; Figure S2D). Moreover, the Matrigel assay indicated that oe‐LINC00261 was capable of decreasing angiogenesis ability of cells compared with Vector, while sh‐LINC00261 increased angiogenesis ability of cells relative to sh‐control (Figure 2E; Figure S2E). Further, Western blot analysis demonstrated that overexpressed LINC00261 led to reduced expression of Brachyury, Ki67, CyclinD1, and VEGF, while opposite trends were observed in response to sh‐LINC00261 (Figure 2F; Figure S2F). The above results supported the proposition that overexpression of LINC00261 could inhibit angiogenesis and cell growth in PC.

### 3.3 LINC00261 regulates the expression of SCP2 through FOXP3

**FIGURE 3:**
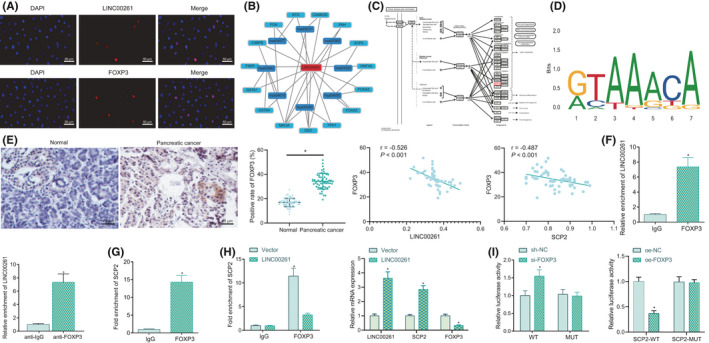
LINC00261 regulates SCP2 through FOXP3. (A) Cell localization of LINC00261 and FOXP3 detected by FISH (200×). (B) Prediction of LINC00261‐related regulatory genes by MEM database. (C) Schematic diagram of SCP2 involvement in PPAR signal pathway. (D) Sequence logo of transcription factor FOXP3 obtained from Jaspar (http://jaspar.genereg.net/). (E) Co‐expression analysis of FOXP3 and LINC00261 in PC tissues by IHC and Pearson correlation analysis of the correlation between LINC00261 and FOXP3, FOXP3 and SCP2 in the PC tissues (400×). (F) The binding of LINC00261 and FOXP3 explored by RIP. G, The binding of FOXP3 and SCP2 assessed by ChIP. (H) The expression of LINC00261, FOXP3 and SCP2 after overexpression of LINC00261 determined with RT‐qPCR and binding of FOXP3 and SCP2 after cells transfected with oe‐LINC00261 detected by ChIP. I, The binding of FOXP3 and SCP2 verified by dual luciferase reporter gene assay. *p < 0.05 vs. that of cells transfected with IgG, oe‐NC or sh‐NC. The experiment was repeated three times

To determine the mechanism by which LINC00261 may be involved in the development of PC, its corresponding localization was observed by FISH assay, which showed most of the LINC00261 and FOXP3 expression was localized in the nucleus of PC cells, and the rest scattered in the cytoplasm (Figure 3A). Then, as predicted by the MEM database, we found that SCP2 was most likely regulated by LINC00261 (Figure 3B). Besides, SCP2 is involved in PPAR pathway (Figure 3C), which has been proven to exert important regulatory functions in PC.^26^, ^27^ Thus, SCP2 may be regulated by LINC00261 in PC. Moreover, a role of FOXP3 in pancreatic ductal adenocarcinoma has been reported.^15^ Therefore, we selected FOXP3 and SCP2 for subsequent analysis. Subsequently, the sequence logo of the transcription factor FOXP3 was further analysed through the Jaspar website (http://jaspar.genereg.net/) as shown in Figure 3D, and the binding site of the target gene SCP2 promoter region was further obtained (Table S3).

Next, IHC results displayed upregulated FOXP3 expression in PC tissues, and Pearson correlation analysis revealed a negative relationship between FOXP3 and LINC00261 as well as between FOXP3 and SCP2 in PC tissues (Figure 3E). RIP assay verified that LINC00261 was able to bind FOXP3 (Figure 3F), and results of the ChIP assay demonstrated that FOXP3 could bind to SCP2 (Figure 3G). Specifically, when LINC00261 was overexpressed, RT‐qPCR displayed that LINC00261 and SCP2 expression was increased significantly, while the expression of FOXP3 decreased significantly after overexpression of LINC00261; meanwhile, ChIP assay results showed reduced SCP2 enrichment in FOXP3 after overexpression of LINC00261 (Figure 3H). AS reflected by dual‐luciferase reporter gene assay, FOXP3 overexpression inhibited SCP2 luciferase activity; FOXP3 silencing increased luciferase activity of SCP2 in PC cells relative to sh‐NC (Figure 3I), suggesting that FOXP3 could specifically inhibit SCP2 expression in PC cells.

### 3.4 LINC00261 represses angiogenesis and cell growth in PC through FOXP3‐madiated SCP2

**FIGURE 4:**
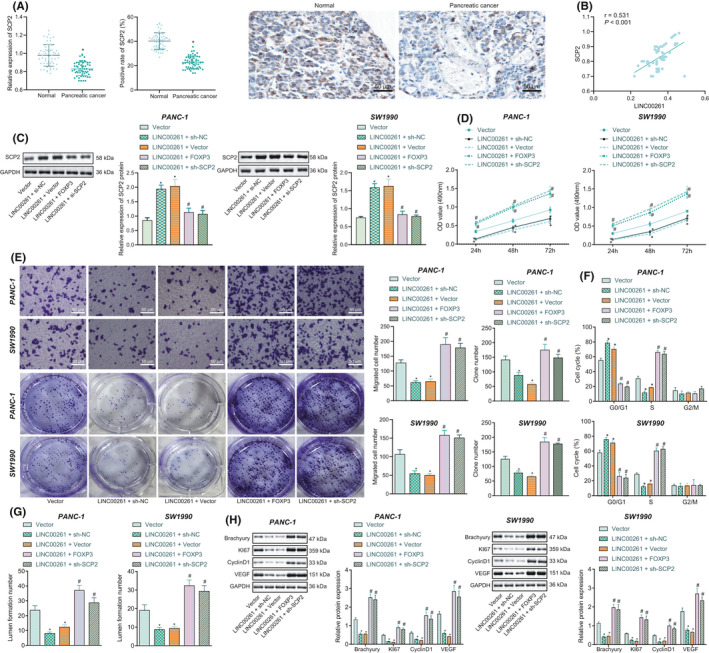
LINC00261 affects biological features of PC cells through FOXP3‐mediated SCP2. (A) The expression of SCP2 in PC tissues and adjacent normal tissues determined by RT‐qPCR and IHC (200×), *p < 0.05 compared with that of adjacent normal tissues, *n* = 57. (B) Pearson correlation analysis of LINC0026 and SCP2 expression in PC tissues. (C) The expression of SCP2 in SW1990 and PANC‐1 cells after treatment with LINC00261, FOXP3 or sh‐SCP2 determined by RT‐qPCR and Western blot analysis. (D) Cell viability of SW1990 and PANC‐1 cells after treatment with LINC00261, FOXP3, or sh‐SCP2 detected by CCK8. (E) The number of cell clones after SW1990 and PANC‐1 cells treated with LINC00261, FOXP3 or sh‐SCP2 assessed by colony formation assay and the statistics. (F) The cycle of SW1990 and PANC‐1 cells after treatment with LINC00261, FOXP3, or sh‐SCP2 measured by flow cytometry. (G) Angiogenesis ability of SW1990 and PANC‐1 cells after treatment with LINC00261, FOXP3, or sh‐SCP2 detected by Matrigel‐based tube formation assay, and statistical graphs. (H) The protein expression of Ki67, Cyclin D1 and VEGF in SW1990 and PANC‐1 cells after treatment with LINC00261, FOXP3 or sh‐SCP2 determined by Western blot analysis, *p < 0.05 compared with cells transduced with Vector, ^#^ *p* < 0.05 compared with cells co‐transduced with LINC00261 + sh‐NC or LINC00261 + Vector. Data are expressed as mean ± standard deviation. One‐way ANOVA was used for statistical analysis among multiple groups. The experiment was repeated three times

We next investigated whether the LINC00261/FOXP3/SCP2 axis participated in angiogenesis and cell cycle regulation in PC. Initial RT‐qPCR and IHC studies showed that SCP2 level was reduced in PC tissues (Figure 4A), and Pearson correlation analysis identified a positive relationship between LINC00261 and SCP2 (Figure 4B). Additionally, elevation of LINC00261 led to elevation of SCP2 expression, which could be reversed by the addition of oe‐FOXP3 or sh‐SCP2 (Figure 4C). Moreover, gain function of LINC00261 led to inhibited proliferation, migration and colony formation abilities, which could be reversed by the treatment of oe‐FOXP3 or sh‐SCP2 (Figure 4D,E). Flow cytometry revealed gain function of LINC00261 arrested more cells in G0/G1 phase, while less cells in S phase, while further treatment of oe‐FOXP3 or sh‐SCP2 could reverse these effects (Figure 4F). The in vitro tube formation test highlighted a reduced angiogenic capacity after overexpression of LINC00261. When co‐treated with LINC00261 + FOXP3 or LINC00261 + sh‐SCP2, the angiogenesis capacity was enhanced (Figure 4G). Further, the protein expression detected by Western blot analysis showed that overexpression of LINC00261 decreased expression of Brachyury, Ki67, CyclinD1, and VEGF, while co‐transduction with LINC00261 + FOXP3 or LINC00261 + sh‐SCP2 upregulated the expression of Brachyury, Ki67, CyclinD1, and VEGF (Figure 4H). In conclusion, LINC00261 affects the angiogenesis and cell cycle of PC cells via the FOXP3/SCP2 axis.

### 3.5 LINC00261 restrains the tumour growth in vivo

**FIGURE 5:**
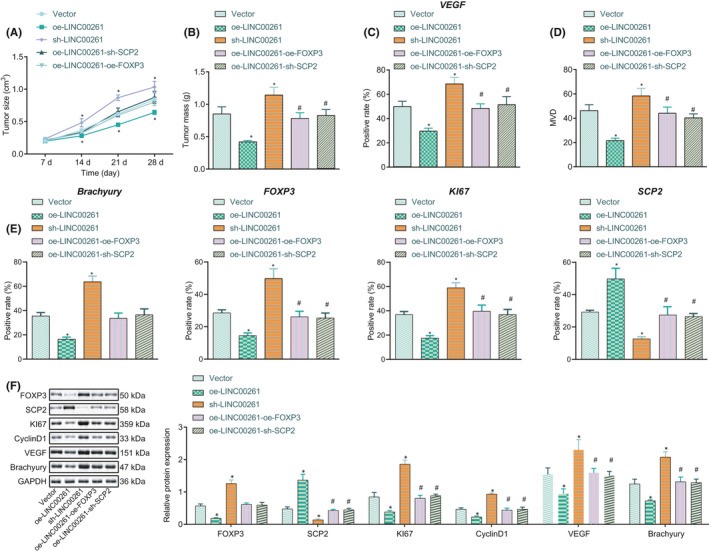
Tumourigenicity in nude mice is inhibited by upregulated LINC00261. (A) Representative images of tumour xenografts and the tumour volume growth curve of nude mice. (B) Tumour weight in nude mice after treatment of oe‐LINC00261. (C) Expression of VEGF in nude mice after treatment with oe‐LINC00261, oe‐FOXP3 or sh‐SCP2 measured by IHC. (D) MVD in nude mice after treatment with oe‐LINC00261, oe‐FOXP3 or sh‐SCP2 measured by IHC. (E and F) Expression of FOXP3, SCP2, Ki67, Cyclin D1, VEGF and Brachyury in nude mice after treatment with oe‐LINC00261 or sh‐LINC00261 measured by IHC and Western blot analysis. *p < 0.05 vs. the mice infected with blank vector. *n* = 5

We constructed a stably transfected PC cell line PANC‐1 expressing oe‐LINC00261, sh‐LINC00261, oe‐FOXP3, sh‐SCP2 or Vector and subcutaneously injected into a tumour model in mice. As depicted in Figure 5A,B and Figure S3 overexpression of LINC00261 reduced the volume and weight of mouse tumours, while knockdown of LINC00261 showed opposite effects. The inhibitory role of overexpressed LINC00261 could be reversed by overexpressing FOXP3 or silencing SCP2. Besides, IHC indicated that the staining site of VEGF‐positive cells was mainly in the cytoplasm of cancer cells. On the other hand, the staining site of CD31 was mainly in the cell membrane and cytoplasm of endothelial cells, with the heavier staining in the cell membrane. Moreover, overexpression of LINC00261 reduced the number of MVD‐positive blood vessels and weakened the VEGF positive expression. However, knockout of LINC00261 had opposite effects, and the inhibitory effect of LINC00261 overexpression could be reversed by upregulating FOXP3 or depleting SCP2 (Figure 5C,D). Further, Western blot analysis showed reduced expression of FOXP3, Ki67, CyclinD1, VEGF and Brachyury in mouse xenograft tumours transduced with oe‐LINC00261, while SCP2 expression was notably upregulated; silencing LINC00261 resulted in the opposite effects, and the effect of LINC00261 overexpression could be abrogated by upregulating FOXP3 or depleting SCP2 (Figure 5E,F). In summary, LINC00261 overexpression impeded the growth of PC tumours in vivo.

## 4 DISCUSSION

Angiogenesis, which is a key factor in several biological processes related to development and progression of cancer, may be regulated by lncRNAs.^28^ We investigated the effects of LINC00261 on the biological process and angiogenesis of PC cells and provided evidence that LINC00261 enhanced SCP2 expression by suppressing the transcription factor FOXP3, thereby inhibiting the angiogenesis and cell cycle change in PC.

We confirmed decreased expression of LINC00261 in PC tissues, as has been reported in a previous study.^29^ In the present study, LINC00261 was further demonstrated to exert an inhibitory role in the ability of proliferation, migration, angiogenesis and tumour formation of PC cells. LINC00261 functions as a tumour suppressor in PC through the miR‐222‐3p/HIPK2/ERK axis.^30^ Dorn et al.^31^ have revealed that LINC00261 downregulation occurred in squamous subtype of pancreatic adenocarcinoma, suggesting LINC00261 as a tumour‐suppressive lncRNA in PC. Moreover, downregulation of LINC00261 was capable of inhibiting cell growth and migration in NSCLC and endometriosis.^32^, ^33^ The inhibitory role of LINC00261 in prostate cancer cell proliferation, migration, as well as angiogenesis, has been demonstrated in relation to the mechanism concerning the GATA6‐mediated DKK3.^34^ These findings are in accord with results of our study, thus indicating that LINC00261 is downregulated in several carcinomas and that its overexpression may play a very important role specifically in PC, thus highlighting LINC00261 as a potential molecular diagnostic marker for target for cancer therapy.

Furthermore, we validated FOXP3 as a target gene of LINC00261, which could be directly inhibited by LINC00261, based on present results from PC‐related datasets and the luciferase assay. FOXP3 is supposed to play a crucial role in the process of regulation of T cell differentiation.^35^ Also, previous work suggests the role of FOXP3 in tumour immunity in cancer patients.^36^ The lymphocyte density of FOXP3^+^ was observed to be related to LNM and further implicated with the development of PC.^37^ Meanwhile, it has been reported that FOXP3 was regulated by lncRNA EGFR‐AS1 and thus is involved in the regulation of NSCLC progression.^17^ Moreover, LINC00261 overexpression decreased the expression of FOXO3 to inhibit the oncogenic phenotype of PC cells in regulation of miR‐552‐5p.^12^ Interestingly, our study showed that LINC00261 could upregulate SCP2 expression by suppressing the expression of FOXP3. As indicated by previous research, SCP2 is able to regulate angiogenesis and tumour migration.^14^ Notably, SCP2 exerts function on vascular inflammation of systemic connective tissue diseases in rats.^38^ Importantly, SCP2 may be especially associated with diseases involving abnormalities in lipid trafficking.^39^ As is widely appreciated, PC is aggressive with dismal prognosis due to its propensity for widespread metastatic disease.^40^ In addition, several research projects have reported an increased de novo lipogenesis in PC cells.^41^, ^42^ Therefore, SCP2 may be involved in the regulation of lipid trafficking in PC cells.

**FIGURE 6:**
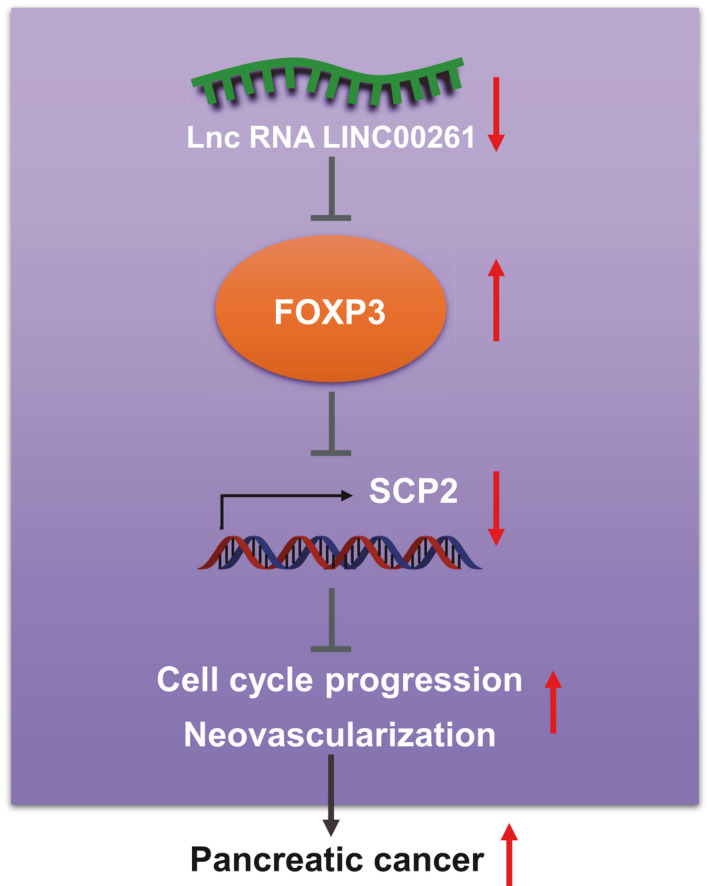
Underlying mechanism of LINC00261 in pancreatic cancer. LINC00261 regulates SCP2 expression in PC by suppressing FOXP3, thus inhibiting angiogenesis and cell cycle progression

In conclusion, we identified that LINC00261 regulates SCP2 expression in PC by suppressing FOXP3, thus inhibiting angiogenesis and cell cycle progression (Figure 6). Our findings present potential clinical therapeutic target for PC treatment, which calls for broader investigations in the clinical setting. However, more samples should be included to better validate the current findings.

## CONFLICTS OF INTEREST

The authors declare that they have no conflicts of interest.

## AUTHOR CONTRIBUTION

**Xuanzeng Pei:** Conceptualization (equal); Data curation (equal); Writing‐original draft (equal). **Jun Zou:** Investigation (equal); Resources (equal); Supervision (equal). **Dan Xing:** Data curation (equal); Formal analysis (equal); Writing‐original draft (equal). **Xiaojun Wu:** Methodology (equal); Software (equal); Visualization (equal). **Shuai Chen:** Project administration (equal); Validation (equal); Writing‐review & editing (equal).

## Supporting information

Figure S1

Figure S2

Figure S3

Table S1‐S3

## Data Availability

Research data are not shared.
