# Supplementary figures and images for: LINC00261 elevation inhibits angiogenesis and cell cycle progression of pancreatic cancer cells by upregulating SCP2 via targeting FOXP3
Source: J Cell Mol Med. 2021 Sep 19;25(20):9826–36. doi: 10.1111/jcmm.16930 (PMC8505824; doi:10.1111/jcmm.16930)

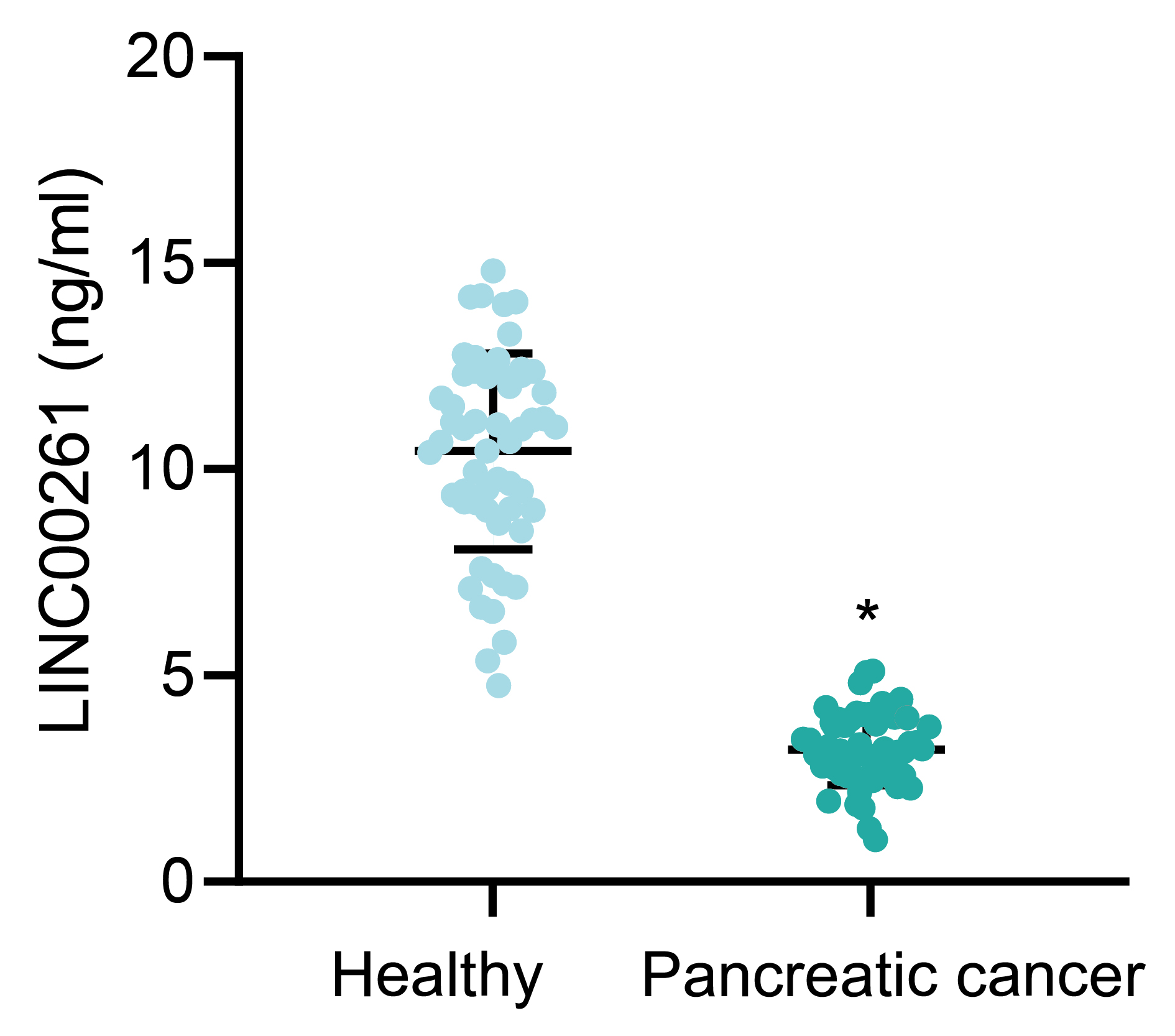

Supplement: Supplementary file 1 — Figure S1 [file JCMM-25-9826-s002.jpg]

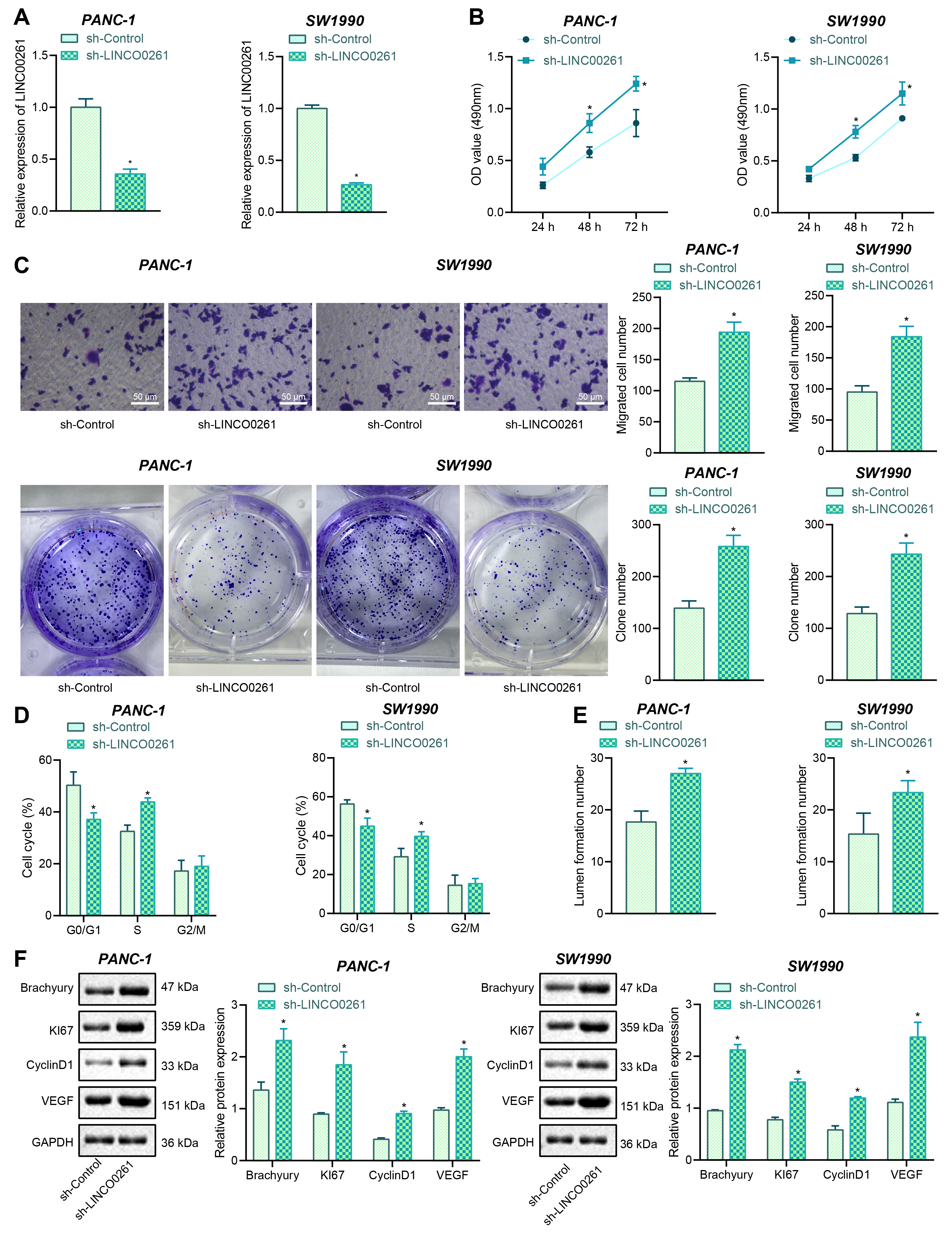

Supplement: Supplementary file 2 — Figure S2 [file JCMM-25-9826-s004.jpg]

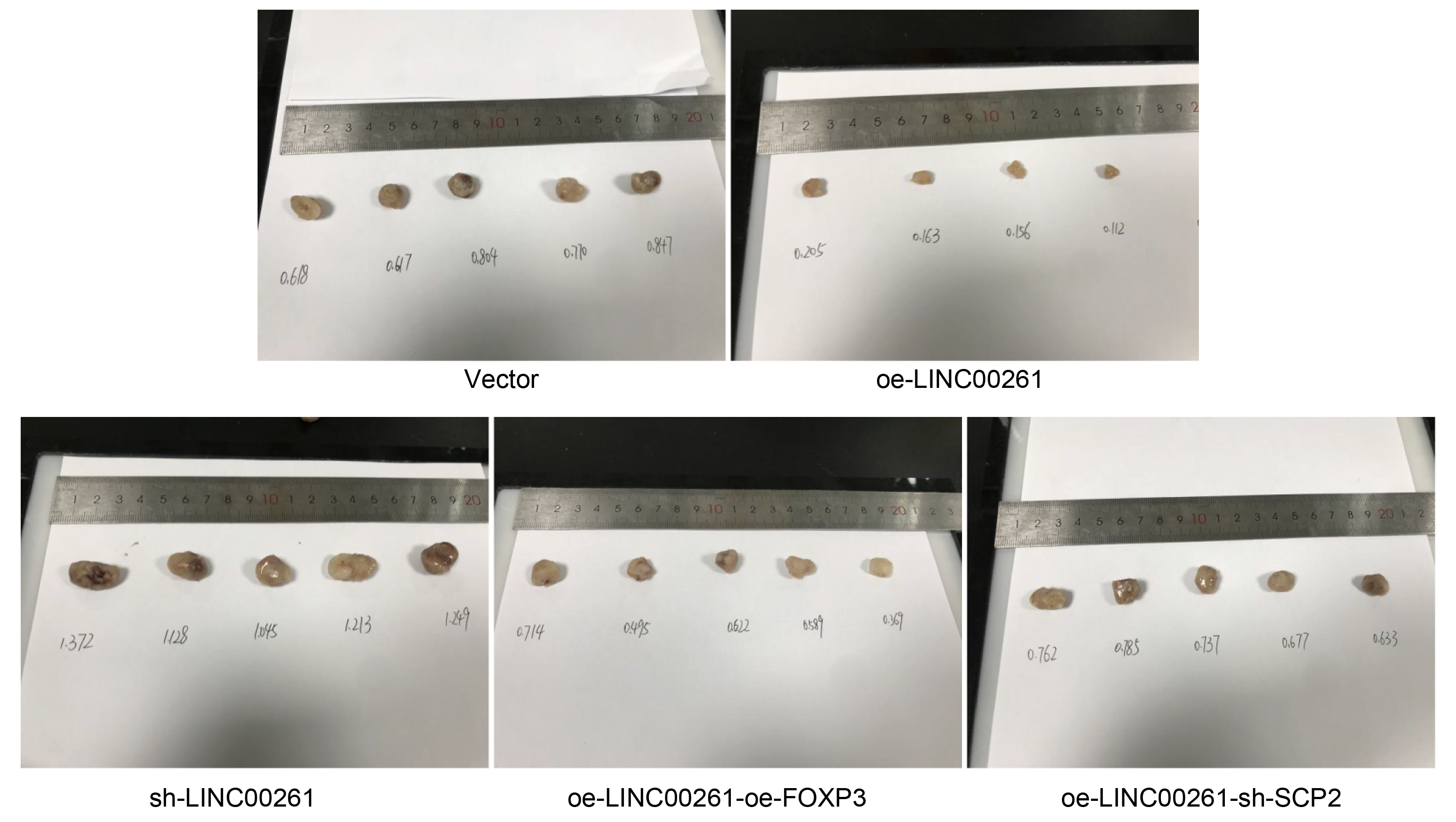

Supplement: Supplementary file 3 — Figure S3 [file JCMM-25-9826-s003.jpg]
