# Supplementary material for: LINC00261 elevation inhibits angiogenesis and cell cycle progression of pancreatic cancer cells by upregulating SCP2 via targeting FOXP3
Source: J Cell Mol Med. 2021 Sep 19;25(20):9826–36. doi: 10.1111/jcmm.16930 (PMC8505824; doi:10.1111/jcmm.16930)
Supplement: Supplementary file 4 — Table S1‐S3 [file JCMM-25-9826-s001.docx]

**Supplementary Table 1** The primer sequences for RT-qPCR

| Gene | Primer sequence |
| --- | --- |
| LINC00261 | F: 5’-GCATTGTGTTGTCAGCCTCA-3’ |
|  | R: 5’-AACCTACAAGTGGTCTGGGG-3’ |
| SCP2 | F: 5’-ATGGGGTTTTCCGGAAGCcCGCCAGTT-3’ |
|  | R: 5’-TCAGAGCTTAGCGTTGCCTGGCTGA-3’ |
| GAPDH | F: 5’-GCACCGTCAAGGCTGAGAAC-3’ |
|  | R: 5’-TGGTGAAGACGCCAGTGGA-3’ |

Note: F, forward; R, reverse; SCP2, sterol carrier protein 2; RT-qPCR, reverse transcription quantitative polymerase chain reaction; GAPDH, glyceraldehyde-3-phosphate dehydrogenase.

**Supplementary Table 2** Relationship between LINC00261 and clinicopathological features of PC patients

| Clinicopathologic characteristics | Cases | LINC00261 | |
| --- | --- | --- | --- |
|  |  | Mean ± SD | P |
| Gender | | | |
| Male | 34 | 0.37 ± 0.05 | 0.153 |
| Female | 23 | 0.35 ± 0.05 |  |
| Age | | | |
| ≤ 60 | 26 | 0.35 ± 0.04 | 0.316 |
| > 60 | 31 | 0.36 ± 0.06 |  |
| Tumor sites | | | |
| Head of pancreas | 33 | 0.37 ± 0.05 | 0.06 |
| Pancreatic body and tail | 24 | 0.34 ± 0.05 |  |
| Tumor diameter (cm) |  |  |  |
| ≤ 2cm | 9 | 0.36 ± 0.09 | 0.452 |
| 2-4cm | 21 | 0.37 ± 0.05 |  |
| > 4cm | 27 | 0.37 ± 0.05 |  |
| Tissue differentiation | | | |
| High | 24 | 0.40 ± 0.04 | 0.014 |
| Medium | 20 | 0.35 ± 0.01 |  |
| Low | 13 | 0.29 ± 0.03 |  |
| Lymphatic metastasis | | | |
| Positive | 30 | 0.33 ± 0.03 | <0.001 |
| Negative | 27 | 0.38 ± 0.06 |  |
| Venous invasion | | | |
| Positive | 24 | 0.34 ± 0.02 | 0.006 |
| Negative | 33 | 0.37 ± 0.06 |  |
| TNM stage | | | |
| I | 18 | 0.38 ± 0.06 | 0.007 |
| II | 27 | 0.36 ± 0.04 |  |
| III | 12 | 0.32 ± 0.03 |  |

**Note:** TNM, tumor node metastasis; LNM, lymph node metastasis; SD, standard deviation.

**Supplementary Table 3** Binding site of FOXP3 on SCP2 promoter in JASPAR website

| Matrix ID | Name | Score | Relative score | Sequence ID | Start | End | Strand | Predicted sequence |
| --- | --- | --- | --- | --- | --- | --- | --- | --- |
| MA0850.1 | FOXP3 | 10.4458 | 00.97850005 | SCP2. | 1479 | 1485 | + | ataaaca |
